# Loop engineering of AtCas9 for effective and broad genome editing

**DOI:** 10.1016/j.cellin.2025.100286

**Published:** 2025-10-22

**Authors:** Yue-Lin Zhang, Dong-Chao Huang, Min Duan, Yu-Ming Zhang, An-Hui Huang, Hao Yin, Ying Zhang

**Affiliations:** a Department of Rheumatology and Immunology, Medical Research Institute, Frontier Science Center for Immunology and Metabolism, Zhongnan Hospital of Wuhan University, Wuhan University, Wuhan 430071, Hubei, China; b Departments of Clinical Laboratory and Department of Urology, Medical Research Institute, Frontier Science Center for Immunology and Metabolism, Zhongnan Hospital of Wuhan University, Wuhan 430071, Hubei, China; c TaiKang Centre for Life and Medical Sciences, TaiKang Medical School, Wuhan University, Wuhan 430071, Hubei, China; d State Key Laboratory of Virology and Biosafety, Wuhan University, Wuhan 430072, Hubei, China

**Keywords:** AtCas9, Loop engineering, Base editing, CRISPR/Cas9, Protein engineering

## Abstract

Efficient genome editing in mammalian cells is essential for CRISPR-based therapeutics. While extensive efforts focused on combining multiple beneficial point mutations to enhance Cas9 activity, the potential of engineering surface-exposed loops remains largely underexplored. Here, we present loop engineering as a streamlined strategy to enhance Cas9 performance. Substituting loops of thermophilic AtCas9 with counterparts from mesophilic Nme1Cas9 generated the AtCas9-Z7 variant, which significantly improves nuclease and base editing efficiency. Biochemical assays showed that Mg^2+^ promotes RNP-DNA interactions, and Z7 maintains high binding affinity under magnesium-limiting condition, a common constraint for Cas9 activity in mammalian cells. Molecular dynamics simulations revealed that Z7 adopts a stable, compact conformation. Importantly, loop engineering can be combined with structure-guided point mutations to further boost activity. The resulting Z7-E78-ABE variant not only achieved a 5.76-fold increase compared to WT AtCas9 and expanded PAM recognition, while enabling editing in primary human T cells, which was not observed with WT AtCas9. Extending this strategy, loop transplantation into GeoCas9 and ThermoCas9 boosted editing efficiency by a median of 14.50-fold and 7.37-fold, respectively, at non-canonical PAMs. Collectively, these results establish loop engineering as a rational and modular approach for Cas9 optimization with therapeutic potential.

## Highlights

• Loop engineering enhances Cas9 activity and broadens PAM compatibility.
• Loop-engineered AtCas9 enables editing in human T cells.
• Loop engineering lowers Mg^2+^dependence, enhancing activity in magnesium-limited eukaryotic cells.
• Loop transplantation generalizes Cas9 optimization across orthologs like GeoCas9 and ThermoCas9.

## Graphical abstract

**Figure:**
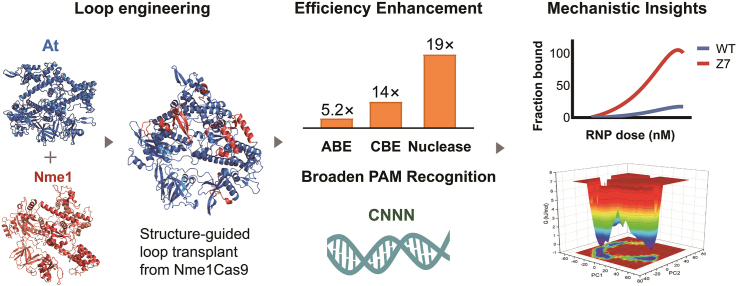

## 1 Introduction

CRISPR/Cas9 system has revolutionized genome editing by enabling precise and programmable manipulation of genomic DNA across diverse organisms (Gao et al., 2023; Liu et al., 2024; Qiu et al., 2022; Wu et al., 2023). Despite its broad utility, many Cas9 orthologs and engineered variants exhibit suboptimal activity in eukaryotic cells, limiting their therapeutic applications (Anzalone et al., 2019; Chen & Liu, 2023; Gasiunas et al., 2020; Gaudelli et al., 2017; Ji et al., 2025; Komor et al., 2016, 2017; Qiu et al., 2022; Reichart et al., 2023; Shi et al., 2022; Wang et al., 2022). Directed evolution and rational design have been employed to enhance Cas9 function (Wang et al., 2021). Directed evolution relies on random mutagenesis combined with selection reporter systems in *E. coli*, yeast, or phage platforms to enrich functional variants (Anzalone et al., 2020; Chen & Liu, 2023; Miller et al., 2020; Qi et al., 2024). Rational design typically focus on introducing mutations to enhance the interactions between the nucleic acid and protein, such as positively charged arginine residues at the nucleic acid-binding surface (Han et al., 2024; Nishimasu et al., 2018; Tong et al., 2025; Zhang, Zhang, et al., 2023) or aromatic ring (Chen et al., 2022) near the base of nucleic acids, at defined structural interfaces. Both approaches have yielded Cas9 variants with improved activity or specificity, while their transferability across orthologs remains challenging due to structural divergence and context dependence.

Loops are flexible structural elements that connect α-helices and β-strands, where they mediate intermolecular interactions and enable conformational transitions. Acting as dynamic hinges between domains, loops play essential roles in regulating enzyme activity, substrate specificity, and structural stability (Arunachalam & Gnanasekaran, 2024; Balasco et al., 2013; Chang & Hsu, 2016; Corbella et al., 2023; Damnjanovic et al., 2014; Fusco et al., 2022; Nestl & Hauer, 2014; Rahban et al., 2022; Zinovjev et al., 2024). Given their functional importance, modifications of loop regions have been used to enhance catalytic efficiency, modulate substrate specificity, and improve thermostability. Cas9 is a multi-domain nuclease that undergoes conformational rearrangements to coordinate target recognition, and catalytic cleavage. Several loop regions within Cas9 have been observed undergo dynamic changes upon sgRNA binding, DNA unwinding and activation (Chen et al., 2017; Jiang et al., 2015, 2016). These features make loops an attractive and underexplored target for systematic engineering to enhance Cas9 performance.

AtCas9 is a thermophilic ortholog characterized by robust thermostability and its relaxed protospacer-adjacent motif (PAM) preference of CNNA and RNNA (N = A/T/C/G, R = A/G). We previously demonstrated that AtCas9 activity is sensitive to DNA topology and, under conditions of DNA underwinding, can achieve PAM-less cleavage in *E. coli* (Shi et al., 2022). However, its limited activity at physiological temperatures restricted its broad application in mammalian cells. Here, we report a loop engineering strategy to improve the activity of AtCas9. Through rational loop redesign and functional screening in mammalian cells, we identified a loop-optimized AtCas9 variant, Z7, incorporating two loop substitutions and one residue substitution in the third loop region. Z7 exhibited a median of 38.97-fold increase in nuclease activity and a median 5.15-fold and 14.42-fold in adenine base editor (ABE) and cytosine base editor (CBE) efficiency, respectively. Mechanistically, Z7 adopts a more stable conformation and strengthens RNP–DNA binding affinity under magnesium-limiting conditions, a common constraint for Cas9 activity in mammalian cells (Eggers et al., 2024). 10.13039/100014337Furthermore, the generalizability of this loop engineering strategy was supported by similar functional gains in other thermophilic Cas9 orthologs, including ThermoCas9 and GeoCas9, highlighting its potential as a modular strategy for Cas9 functional enhancement.

## 2 Results

### 2.1 Loop engineering enhances the editing efficiency of thermophilic AtCas9

**Fig. 1:**
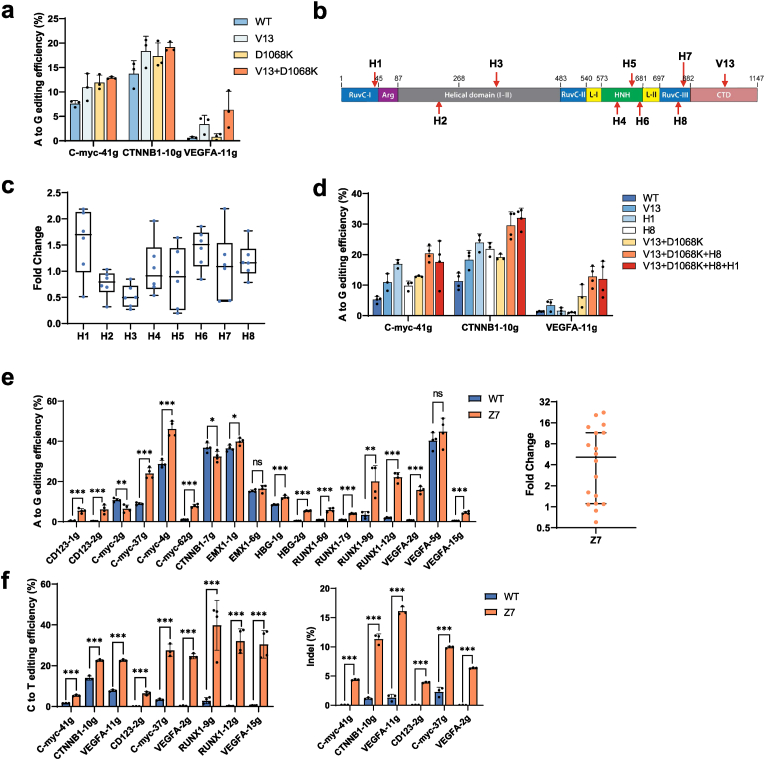
**Loop engineering enhances the editing efficiency of thermophilic AtCas9.** (a) Editing efficiencies of indicated variants at three genomic sites in HEK293T cells. (b) Illustration of AtCas9 domains with engineered loops marked with red arrows. (c) Box plots showing fold changes in editing efficiency of loop engineered AtCas9-ABE8e mutants at six genomic sites in HEK293T cells. H1-H8 indicates different loops in AtCas9. (d) Editing efficiencies of combination loop engineering of AtCas9-ABE8e. (e-f) Editing efficiency of wildtype and the loop engineered AtCas9-Z7 mutant (V13+D1068K+​H8) at genomic sites in HEK293T cells. Genome editing was performed using three different editor combinations: AtCas9-ABE8e, AtCas9-BE3 (rAPOBEC1-XTEN-nCas9-UGI), and AtCas9 nuclease. For (a), (d-f), bars represent mean ± SD from three or four independent biological replicates. Each dot represents one biological experiment. For (e) and (f), statistical significance was determined by unpaired *t*-test (∗*p* ​< ​0.033, ∗∗*p* ​< ​0.002, ∗∗∗*p* ​< ​0.001). For (c) and summary data in (e), box plots show the median and interquartile range, and horizontal lines indicate the median with 95​% CI, respectively, with each dot representing the fold change observed at a single genomic target.

AtCas9 is a typeⅡ-C thermophilic Cas9 that exhibits optimal cleavage activity at 55 °C but shows reduced activity at 37 °C (Shi et al., 2022) (Fig. S1a). Unlike mesophilic allosteric proteins, which often rely on flexible loops for conformational changes, thermophilic proteins typically feature rigid structural cores and constrained loops, requiring elevated temperatures for activation. While the core regions are generally conserved between thermophilic and mesophilic Cas9, their loop regions show low sequence conservation and differ significantly in length and physicochemical properties (Fig. S1b). Given the potential role of loop regions in modulating protein flexibility and temperature responsiveness, we hypothesized that engineering the loop regions of AtCas9 could lower its allosteric activation threshold and enhance its activity at physiological temperatures. To test this, we performed structural comparisons with the mesophilic homolog Nme1Cas9 (Ibraheim et al., 2018; Lee et al., 2016; Sun et al., 2019; Zhang et al., 2015), which shares 37% sequence identity and functions optimally at 37 °C (Sun et al., 2019). The comparison revealed that highly conserved core elements (α-helix and β-sheets), but substantial divergence in loop regions (Fig. S1b). To identify functionally relevant loops, we aligned Nme1Cas9 structures in the ribonucleoprotein (RNP) (PDB: 6JDQ) and active (PDB: 6JDV) states and mapped the loops undergoing conformational rearrangements onto AtCas9. We first focused on the C-terminal domain (CTD), which mediates sgRNA and DNA interactions. The loop spanning residues 915–933 (ELKLTKDGEIKDYFRPEDD) in AtCas9 was replaced with the corresponding Nme1Cas9 sequence (866–881, QLKLKDLEKMVNRERE), generating the V13 variant (Fig. S1c). Fusion of V13 with AtCas9-ABE8e enhanced base editing activity at three genomic loci in HEK293T cells, with an average 2.81-fold increase over wild-type AtCas9 (Fig. 1(a)). Structure modeling indicated that the engineered V13 loop forms a short α-helix stabilized by salt bridges and hydrogen bonding (notably between E919/R924 and Y937), whereas the native loop is disordered (Fig. S1d). This structural transition likely contributes to improved loop rigidity and overall conformational stability.

To assess whether loop engineering could be synergistically combined with rational design and further enhance editing efficiency, we introduced a charge-reversal mutation at residue 1068, substituting the negatively charged aspartate with a basic lysine (D1068K). This mutation resulted in an average 1.39-fold increase in AtCas9-ABE8e editing efficiency at three genomic loci compared to wild-type AtCas9 (Fig. 1(a)). Structural modeling indicates that the D1068K substitution enables the loop to move closer to the DNA duplex, allowing the lysine side chain to form hydrogen bonds with the target strand (NTS) (Fig. S1e). Notably, combining the D1068K mutation with the V13 loop substitution led to a further increase in editing efficiency, resulting in an average 4.55-fold improvement over the WT (Fig. 1(a)), highlighting the compatibility and additive effect of these two strategies.

Inspired by the initial success of loop engineering, we next extended this strategy to additional regions, generating variants H1-H8 (Fig. 1(b) and Fig. S1f). Among these, H1 and H8 exhibited enhanced base editing activity, with median increases of 1.70-fold and 1.16-fold, respectively, across three tested sgRNAs (Fig. 1(c) and Fig. S1g). Incorporating the H8 loop into the V13+D1068K background yielded the Z7 variant, which further improved editing efficiency, achieving 3.88-fold, 2.61-fold, and 9.48-fold increases at *C-myc*-41g, *CTNNB1*-10g, and *VEGFA*-11g loci, respectively (Fig. 1(d)). To further characterize At-Z7, we evaluated its performance across adenine base editing (ABE), cytosine base editing (CBE), and nuclease-mediated DNA cleavage. At-Z7-ABE demonstrated improved activity, with up to a 5.15-fold median increase across 18 target sites (Fig. 1(e)). Similarly, At-Z7 significantly enhanced CBE efficiency, showing a median 14.42-fold increase, and improved nuclease activity by 38.97-fold across multiple loci (Fig. 1(f)).

Structural simulations revealed that the H8 loop is positioned near the end of the hybridized strand and increases the local electrostatic potential, rendering the protein surface more positively charged (Fig. S1h). This change likely alters the surface charge distribution near the nucleic acid binding interface, enhancing electrostatic attraction to DNA. Collectively, these results demonstrate that loop engineering effectively enhances AtCas9 activity in eukaryotic cells and acts synergistically with rationally designed mutations to improve the performance of three different genome editors.

### 2.2 Z7 enables efficient RNP-DNA binding under magnesium-restricted conditions

**Fig. 2:**
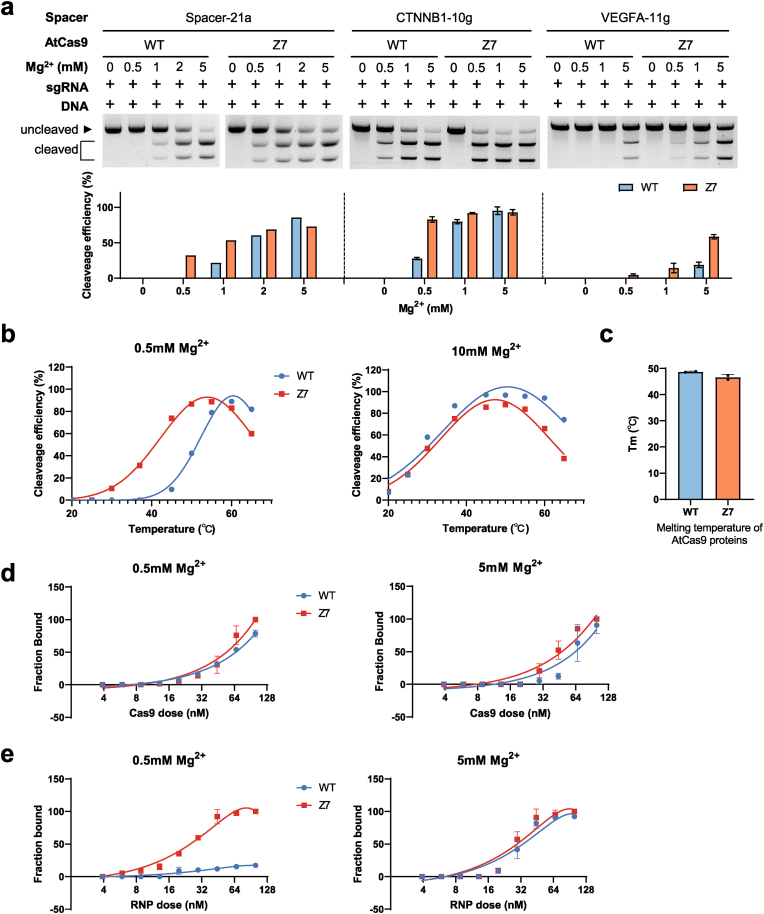
**Z7 enhances DNA binding and cleavage activity of AtCas9 at low magnesium condition.** (a) *In vitro* cleavage assay of WT and Z7 proteins with different concentrations of Mg^2+^ (0.5, 1, 2, 5 mM). Three target loci (*Spacer-21a*, *CTNNB1-10g*, and *VEGFA-11g*) were tested. (b) Temperature-dependent cleavage efficiency of WT and Z7. (c) Thermal shift analysis of melting temperatures of the AtCas9 proteins. (d-e) EMSA analysis of binding affinities between Cas9 variants and sgRNA (d), or Cas9 RNP complexes with DNA substrates (e), under the indicated magnesium concentrations.

To investigate how loop engineering enhances AtCas9 activity, we purified AtCas9-WT and AtCas9-Z7 for biochemical analysis. Despite AtCas9-Z7 exhibiting consistent higher genome editing efficiency *in vivo*, the *in vitro* cleavage activity varies across target sites (Fig. S2a). One key difference between *in vitro* cleavage and the intracellular mammalian environment is the concentration of free magnesium ions (Mg^2+^), which critically influences DNA binding and catalytic activity of typeⅡ-C Cas9 proteins (Eggers et al., 2024; Nguyen et al., 2024; Raper et al., 2018). While biochemical reaction buffer contains 10 mM free Mg^2+^, physiological Mg^2+^ concentrations in mammalian cells are much lower (0.1–1 mM) (Fatholahi et al., 2000; Raju et al., 1989). It is possible that loop engineering reduces the Mg^2+^ dependency of AtCas9. To test this, we performed the *in vitro* cleavage activity of AtCas9-WT and Z7 at 0.5, 1, and 5 mM MgCl_2_ using two mammalian genomic targets (*CTNNB1-10g* and *VEGFA-11g*) and one original CRISPR locus (*Spacer -21a*). At 5 mM Mg^2+^, both showed comparable cleavage activity. However, at 0.5 mM Mg^2+^, a physiologically relevant concentration, AtCas9-WT showed markedly reduced activity, whereas Z7 maintained efficient cleavage (Fig. 2(a)). These results suggest that loop engineering reduces the Mg^2+^ dependency of AtCas9-Z7, enabling efficient genome editing under magnesium-limiting conditions in mammalian cells.

We next investigated whether the Z7 mutation alter temperature sensitivity under magnesium-limited conditions. At high Mg^2+^ conditions, AtCas9-Z7 and AtCas9-WT exhibited similar temperature optima, with peak cleavage efficiency observed at 55 °C (Fig. 2(b) and Fig. S2b). However, under Mg^2+^ restrictive conditions, we observed a notable shift in the optimal cleavage temperature: while AtCas9-WT required elevated temperatures (up to 60 °C) to achieve maximal activity and showed minimal activity at 37 °C, Z7 retained detectable activity at 37 °C and exhibited peak activity at a lower temperature than WT (Fig. 2(b) and Fig. S2b). This shift suggests that loop transplantation from a mesophilic ortholog may reduce the allosteric activation threshold of AtCas9, enabling efficient cleavage under physiological temperatures and magnesium concentrations. To further assess whether the altered thermal dynamics was due to a global protein stability, we performed thermal shift assay. Z7 displayed a melting temperature (*T*_m_) of 46.6 °C, comparable to 48.7 °C for the wild type (Fig. 2(c)), indicating that its overall thermostability remains largely intact. Together, these results demonstrate that while AtCas9-Z7 remains thermophilic properties, it exhibits markedly expanded temperature tolerance and significantly improved activity at physiological temperatures under magnesium-limited conditions.

Mg^2+^ is an essential cofactor for Cas9-mediated DNA cleavage. For AtCas9, Mg^2+^ is required not only for catalysis within the HNH and RuvC domains, but also for stabilizing interactions between the protein and sgRNA (Shi et al., 2022). To further dissect the step at which loop engineering contributes to enhanced activity, we performed electrophoretic mobility shift assays (EMSA) to evaluate the binding kinetics of Cas9-gRNA and RNP–DNA complexes, respectively. Z7 exhibited sgRNA binding affinity comparable to that of WT protein (Fig. 2(d) and Fig. S2c). However, under low Mg^2+^ condition, Z7 maintained robust DNA binding, whereas AtCas9-WT displayed minimal DNA-binding capacity (Fig. 2(e) and Fig. S2d). These findings suggest that Z7 enhances genome editing activity by stabilizing the RNP-DNA complex under magnesium-limiting conditions, thereby facilitating genome targeting in mammalian cells.

### 2.3 Molecular dynamics (MD) simulation of AtCas9-Z7 ternary complex

**Fig. 3:**
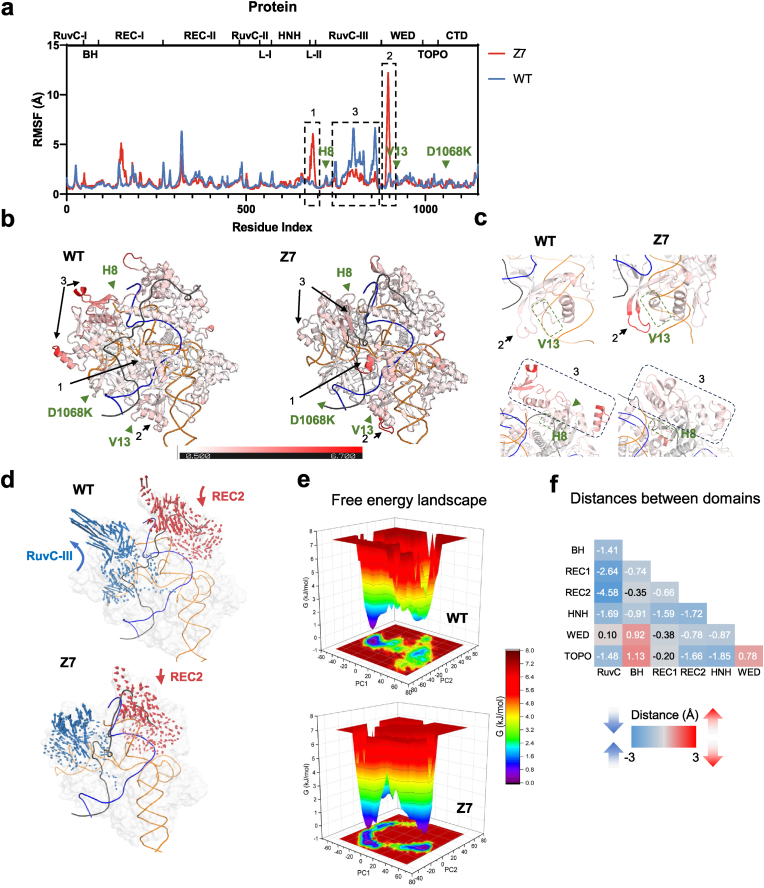
**MD simulations reveal that loop engineering enhances structural stability and promotes a catalytically competent conformation of AtCas9.** (a) RMSF profiles of WT AtCas9 (blue) and the loop-engineered variant Z7 (red), with engineered loops marked by green arrows. Domain boundaries are indicated above. Boxed regions highlight altered flexibility in L2 and WED (increased) and RuvC-Ⅲ (decreased). (b) RMSF values mapped onto the protein structure, with colors ranging from white (rigid) to red (flexible), illustrating the overall flexibility distribution. Regions with altered flexibility in L2, WED (increased), and RuvC-Ⅲ (decreased) are marked by black arrows. (c) Detailed comparison of RMSF values between WT and Z7 variants in the boxed regions. (d) Motion trajectory of RuvC-Ⅲ and REC2 domain during MD simulations. (e) Free energy landscapes based on principal component analysis (PC1 and PC2) from 1 μs simulations. (f) Interdomain distances measured in minimum energy conformations.

To elucidate how loop engineering enhances AtCas9-sgRNA-DNA binding affinity, we performed 1 μs MD simulations at 37 °C on ternary complexes of WT AtCas9 and the loop-engineered variant Z7, both modeled in the catalytically “ready-to-cleave” conformation with the HNH domain positioned near the cleavage site. Backbone root mean square deviation (RMSD) analysis confirmed that both systems reached equilibrium after 200 ns (Fig. S3a). We then calculated per-residue root mean square fluctuation (RMSF) over the equilibrated trajectory (200–1000 ns) to assess local flexibility. Z7 showed slightly lower global RMSF (1.23 Å) compared to WT (1.29 Å), indicating enhanced overall stability. The engineered regions (H8, V13, and D1068K) did not exhibit notable local flexibility changes, whereas their neighboring regions displayed distinct flexibility differences between Z7 and WT (Fig. 3(a)). The boxed region 1 and region 2, located in the L2 linker and WED domain, exhibited increased flexibility (Fig. 3(a) and (b)). As L1 undergoes conformational rearrangement to reposition the HNH domain toward the scissile phosphate, and L2 coordinates this transition and facilitates RuvC activation, it is possible that the increased flexibility in L2 may facilitate allosteric transitions during Cas9 activation. In contrast, the boxed region 3 had reduced flexibility across the RuvC-Ⅲ domain (Fig. 3(b)), and this local rigidification may strengthen RNP-DNA engagement (Fig. 3(c)). Further analysis revealed that the engineered H8 and V13 loops formed additional stable hydrogen bonds and salt bridges with nucleic acids, thereby enhancing stabilization of the protein-RNA-DNA ternary complex (Fig. S3b). To investigate how stabilization of the RuvC-Ⅲ domain influences the conformation of the Cas9, we performed essential dynamics analysis based on principal component 1 (PC1). Compared to the outward opening observed in the WT complex, the RuvC-Ⅲ domain in Z7 moves inward toward the PAM-distal end of the guide-target heteroduplex (Fig. 3(d)). This inward movement, in coordination with the REC2 domain, forms a clamp-like configuration that anchors the PAM-distal duplex, facilitating the conformational transition of the HNH domain into its catalytically active state.

To characterize conformational energy landscapes, we constructed three-dimensional free energy landscapes using principal component analysis (PCA). Compared to WT, Z7 occupied a narrower and deeper free energy basin with two distinct energy minima, indicating reduced conformational heterogeneity and a more stable dominant conformation (Fig. 3(e)). Structural analysis of the minimum energy conformations revealed significantly shorter inter-domain distances in Z7, particularly between the RuvC domain and the BH, REC1, and REC2 domains, ranging from 1.41 Å to 4.58 Å (Fig. 3(e)). This compaction suggests a structural transition toward a more catalytically competent conformation (Fig. 3(f)). Collectively, these simulations support that Z7 adopts a more stable and compact structural ensemble, potentially underlying its enhanced genome editing activity through intrinsic conformational optimization.

### 2.4 Enhancing protein-nucleic acid affinity to improve editing efficiency

**Fig. 4:**
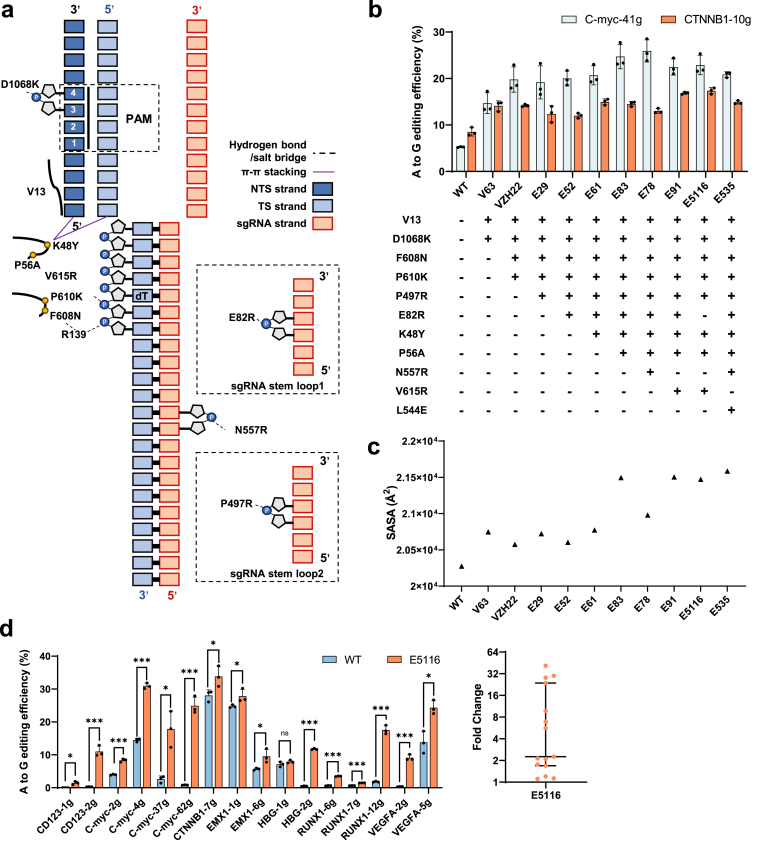
**Structure-guided protein engineering improves the editing efficiency of AtCas9.** (a) Schematic representation of sgRNA: DNA target recognition by AtCas9 variants. Residue substitutions in the E535 and E5116 variants are highlighted. (b) Adenine base editing efficiencies of AtCas9 mutants at *C-myc-41g* and *CTNNB1-10g* target sites. (c) Solvent-accessible surface area (SASA) analysis of interactions between different AtCas9 variants and nucleic acids, reflecting changes in protein–nucleic acid interface accessibility. (d) (left) A-to-G editing efficiencies of wildtype and AtCas9 E5116 mutant. (right) Median fold change of editing efficiency. Data were normalized to WT AtCas9-ABE. For (b) and (d), data are mean ± SD of *n* = 3 biologically independent experiments. Statistical significance was determined by unpaired *t*-test (∗*p* ​< ​0.033, ∗∗∗*p* ​< ​0.001). Each dot represents one biological experiment.

Electrostatic interactions, particularly between the negatively charged phosphodiester backbone of nucleic acids and positively charged residues on Cas9, play a critical role in stabilizing RNP assembly and promoting efficient DNA targeting and cleavage (Han et al., 2023, 2024; Shi et al., 2022; Yan et al., 2024). To enhance AtCas9-nucleic acid interactions, we screened 16 residues located proximal to DNA or sgRNA in the AtCas9 ternary structure using two strategies: arginine scanning to introduce positive charge and polarity-based substitutions guided by the Nme1Cas9 ortholog. Six mutations, F608N, P610K, P497R, E82R, N557R, and V615R, exhibited improved A-to-G base editing efficiencies (Fig. S4a). Structural modeling suggests that F608N and P610K, located within a loop region of the HNH domain, stabilize the heteroduplex through indirect hydrogen bonding and electrostatic interactions (Fig. 4(a)). V615R moves closer to the DNA target strand upon allosteric activation, while N557R contributes to heteroduplex stabilization during the active state. E82R and P497R enhance sgRNA binding by reinforcing salt bridge formation at stem loops 1 and 2, respectively (Fig. 4(a)). To further promote unwinding, aromatic residues (W, Y) were introduced at position K48 to exploit π–π stacking interactions (Chen et al., 2022). Among these, K48Y markedly boosted editing efficiency by stabilizing PAM duplex unwinding (Fig. S4a).

Progressive accumulation of compatible mutations resulted in gradual improvements in editing efficiency (Fig. 4(b)) and an increase in solvent-accessible surface area (SASA), suggesting enhanced Cas9–nucleic acid binding (Fig. 4(c)). However, systematic combination of multiple beneficial mutations revealed a threshold beyond which further accumulation impaired editing activity (e.g., E93, E103–E107), potentially due to unfavorable epistatic interactions (Fig. S4a). To mitigate, we selected residues E82 and L544 for saturation mutagenesis, as structural analyses suggested that these positions might introduce local strain or unfavorable epistasis with previously introduced modifications. The goal was to identify substitutions that could alleviate such conflicts and improve overall stability and editing consistency. However, these substitutions failed to enhance editing efficiency, prompting us to remove the mutations. This led to the generation of the E5116 and E535 mutants (Fig. S4b). Further evaluation of editing efficiency at *VEGFA-1g* and *VEGFA-11g* indicated that E5116 exhibited superior performance over E535 (Fig. S4c). The final optimized variant, At-E5116, which carries eight synergistic mutations (K48Y, P56A, P497R, F608N, P610K, V615R, V13, D1068K), achieved an average editing efficiency of 15.08% across 16 additional loci, representing up to a 2.25-fold median improvement (Fig. 4(d)).

### 2.5 The combination of loop transplant and point mutations further boosted activity

**Fig. 5:**
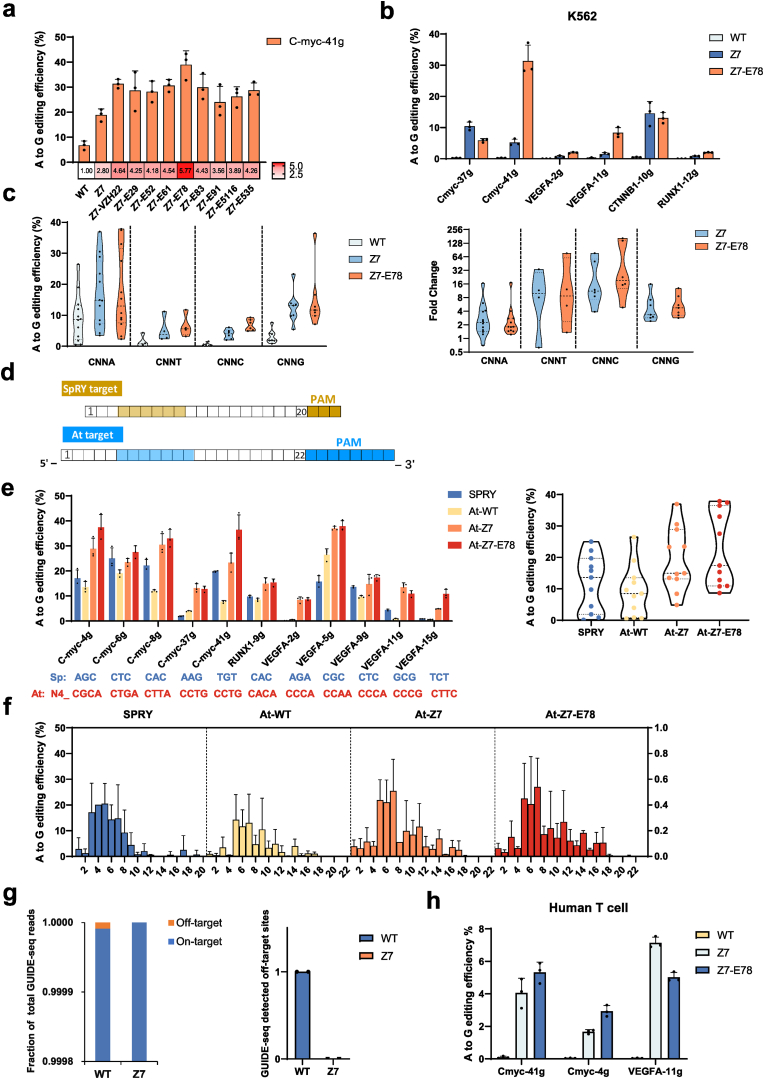
**Evaluation of base editing efficiency and editing window for AtCas9 mutants.** (a) A-to-G editing efficiencies of combinational variants incorporating loop engineering and structure-guided rationale design. (b) A-to-G editing efficiencies of indicated variants in K562 cells. (c) (left) A-to-G editing efficiencies of Z7-ABE and Z7-E78-ABE variants targeting indicated PAM sequences in HEK293T cells. (right) Median fold change of editing efficiency. Data were normalized to WT AtCas9-ABE. (d) Schematic representation of spacer-matched site design used for comparing editing efficiency between SPRY-ABE and AtCas9 variants. (e) Comparative analysis of editing efficiency at spacer-matched sites for SPRY-ABE, AtCas9-WT-ABE, AtCas9-Z7-ABE, and AtCas9-Z7-E78-ABE. (f) Editing window and base editing activity comparison for SPRY-ABE, AtCas9-WT-ABE, AtCas9-Z7-ABE, and AtCas9-Z7-E78-ABE. (g) (Left) Fraction of on-target integration reads over total reads for AtCas9-WT and AtCas9-Z7. (Right) Number of off-target sites for AtCas9-WT and AtCas9-Z7. (h) Editing activity of AtCas9-ABE variants in primary human T cells. For (a-b), (e), (h), bars represent mean ± SD from three independent biological replicates. Each dot represents one biological experiment. For (c) and combined data in (e), bars represent median with 95% CI. For (f), mean ± SD derived from 20 different spacers.

To investigate the synergistic effect between loop engineering and structure-based engineering, we selected several key mutations known to enhance protein nucleic acid affinity and integrated them into the Z7 framework. The editing efficiency of these variants was assessed at the endogenous *C-myc-41g* and all nine combinations tested exhibited substantial improvements (Fig. 5(a)). Among them, the Z7-E78 variant, which harbors eight point-mutations and two loop transplants (Fig. S5a), exhibited the highest efficiency, representing a 5.76-fold improvement over WT, and a 2.06-fold increase over Z7 (Fig. 5(a)). Further comparison of the Z7 and Z7-E78 mutants in K562 cells across six endogenous loci revealed that the Z7 conferred improved editing activity at all sites, while the addition of the E78 mutations further enhanced editing efficiency, resulting in a 2.65-fold median increase relative to Z7 (Fig. 5(b)). These results demonstrate that rationally designed structural mutations are compatible with loop engineering and can be integrated synergistically to further boost editing performance.

AtCas9 has broad PAM preference showing high affinity towards 5′-N4CNNA-3′ and 5′-N4RNNA-3′ sequence (*R* = A/G) (Shi et al., 2022). To evaluate the performance of Z7 and Z7-E78 against different PAM sequences, we measured their editing efficiencies across 29 genomic sites bearing 5′-N4CNNN-3′ PAMs in HEK293T cells. Among the 12 sites with CNNA PAMs, WT averaged 9.17%, compared to 17.23% and 17.49% for Z7 and Z7-E78, corresponding to 2.25- and 1.81-fold enhancements. At sites with suboptimal CNNB PAMs (B = T/C/G), WT activity was markedly reduced, with average efficiencies of 1.50% (CNNT), 0.49% (CNNC), and 3.18% (CNNG). In contrast, Z7 and Z7-E78 exhibited robust improvements, corresponding an overall improvement of 8.07-fold and 8.57-fold at the CNNB PAMs, respectively (Fig. 5(c) and Fig. S5b). These findings highlight that Z7 and Z7-E78 not only enhance editing efficiency but also broaden the targeting scope of AtCas9-ABE.

We next compared the performance of Z7-ABE and Z7-E78-ABE with SpRY-ABE, an engineered variant previously reported to possess broadened PAM compatibility (Walton et al., 2020) (Fig. 5(d)). Across 11 genomic sites, Z7-ABE exhibited comparable editing efficiency to SpRY-ABE, whereas Z7-E78-ABE showed superior performance than SpRY-ABE (Fig. 5(e)). Further analysis of the editing windows revealed distinct differences among the variants. SpRY-ABE8e displayed a relatively narrow editing window, predominantly spanning positions 3–7 of the protospacer. In contrast, both Z7-ABE and Z7-E78-ABE retained broader editing windows, extending from positions 5–11, consistent with that of WT (Fig. 5(f)). These findings underscore the advantage of Z7 and its derivatives in expanding the flexibility of base editing, not only through enhancing PAM compatibility but also by preserving a wider editing range.

To evaluate whether loop engineering compromises genome-wide specificity, we performed GUIDE-seq profiling of AtCas9-WT and the engineered variant AtCas9-Z7 using the CTNNB1-7g spacer. Off-target detection was conducted with a mismatch threshold of 8 in two independent biological replicates. AtCas9-WT consistently produced the one off-target site in replicates, whereas AtCas9-Z7 showed no detectable off-targets in two replicates (Fig. S6). Notably, the OT reads detected in WT accounts for <0.02% of total reads (Fig. 5(g)), further indicating that AtCas9 is a high-fidelity nuclease. Importantly, the engineered Z7 variant retains high target specificity while exhibiting enhanced on-target editing efficiency, indicating that loop engineering does not increase off-target effects.

To extend these findings to a clinically relevant context, we next assessed AtCas9-ABE-WT, Z7, and Z7-E78 in primary human T cells by electroporating Cas9 mRNAs together with chemically modified sgRNAs targeting *Cmyc-g4*, *Cmyc-g41*, and *VEGFA-g11*. Deep sequencing revealed negligible editing by WT, whereas Z7 and Z7-E78 achieved 2%–7% efficiency across the tested sites (Fig. 5(h)). These results highlight that the engineered AtCas9 variants not only maintain high specificity and efficiency but also hold potential for therapeutic genome editing applications.

### 2.6 Loop-engineering strategy improved activities for other thermophilic Cas proteins

**Fig. 6:**
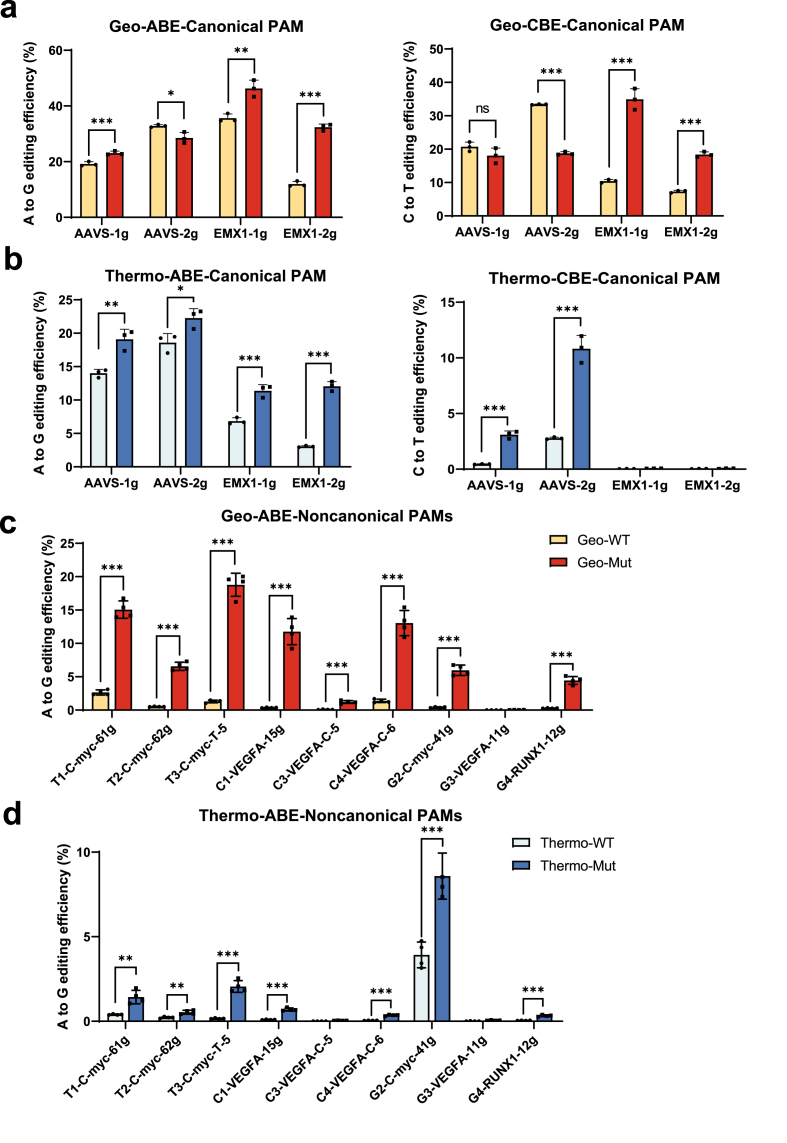
**Editing efficiencies ofloop-engineeredGeoCas9 and ThermoCas9 mutants.** (a-b) Editing efficiency of wildtype and the loop engineered GeoCas9 mutant and ThermoCas9 mutant (H8+V13) in HEK293T cells using the canonical PAM CTAA. The H8 and V13 loops were replaced with the corresponding sequence from Nme1Cas9. (c-d) Editing efficiencies of wild-type and loop-engineered GeoCas9 and ThermoCas9 ABEs were measured at nine endogenous sites containing non-canonical PAMs (CNNT, CNNC, CNNG). For (a-d) data are mean ± SD of *n*= 3 or 4 biologically independent experiments. Statistical significance was determined by unpaired *t*-test (∗*p* ​< ​0.033, ∗∗*p* ​< ​0.002, ∗∗∗*p* ​< ​0.001).

To investigate the broader applicability of the loop engineering strategy, we examined whether loop transplant could be applied to other Cas9 orthologs. Specifically, we selected GeoCas9 (Chen et al., 2025; Eggers et al., 2024; Harrington et al., 2017) and ThermoCas9 (Mougiakos et al., 2017; Shen et al., 2024), two thermophilic nucleases with optimal temperatures of 55 °C and 60 °C, respectively. Sequence alignment with Nme1Cas9 and AtCas9 identified corresponding loop regions for substitution (Fig. S7). We introduced the H8 and V13 loop regions from Nme1Cas9 into GeoCas9 and ThermoCas9, and assessed their activity in HEK293T cells. At four endogenous sites bearing canonical CTAA PAM in the *AAVS1* and *EMX1* loci, loop-engineered variants displayed modest but reproducible improvements in editing efficiency (Fig. 6(a) and (b)). When targeting nine addition loci harboring non-canonical PAMs (CNNT, CNNC, and CNNG), loop-engineered variants showed significantly higher editing efficiencies than their wild-type counterparts, with GeoCas9 variants reaching 5%–20% efficiency (Fig. 6(c) and (d)). These results suggest that ortholog-specific loop optimization is particularly beneficial under suboptimal PAM conditions and represents a generalizable strategy across Cas9 orthologs.

## 3 Discussion

Loops are dynamic structural elements and modulation of loop properties has been shown to alter enzymatic properties (Arunachalam & Gnanasekaran, 2024; Balasco et al., 2013; Chang & Hsu, 2016; Corbella et al., 2023; Damnjanovic et al., 2014; Fusco et al., 2022; Nestl & Hauer, 2014; Rahban et al., 2022; Zinovjev et al., 2024). However, loops in Cas9 remain an underexplored target in protein engineering, particularly those located distal to the active sites, making them less intuitive candidates for conventional rational design. In this study, we developed a loop engineering strategy to enhance genome editing efficiency of the thermophilic AtCas9. Guided by structural alignment with its mesophilic homolog Nme1Cas9, we identified loops in AtCas9 that undergo conformational changes during allosteric activation. Replacing these loops with their Nme1Cas9-derived counterparts generated the Z7 variant, which exhibited significantly improved nuclease and base editing activity in HEK293T and K562 cells. Moreover, we demonstrate that loop engineering strategy can be synergistically combined with rationally screened point mutations to further enhance editing outcomes. This is exemplified by the Z7-E78-ABE, a variant that incorporates loop substitutions and targeted residue changes, resulting in not only improved editing efficiency but also expanded PAM compatibility from CNNA to CNNN. Notably, Z7-E78-ABE achieves higher editing activity than the PAM-relaxed SpRY editor.

Mechanistically, we found that the loop-engineered Z7 variant enhances genome editing efficiency in mammalian cells by stabilizing RNP-DNA binding under magnesium-restricted conditions, which are physiologically relevant in cell. MD simulations further revealed that Z7 adopts a more compact and thermodynamically stable conformation compared to wild-type AtCas9. This structural stabilization likely contributes to its improved activity in cellular environments with limited free Mg^2+^. Although the melting temperature of Z7 remains comparable to wild type, the local energetic barriers to conformational activation may be reduced, facilitating allosteric transitions required for catalysis. Supporting this view, a GeoCas9 variant bearing wedge domain mutations exhibited enhanced DNA unwinding under magnesium-restricted conditions (Eggers et al., 2024), suggesting a broader principle that reducing magnesium dependency through direct evolution or loop engineering offers a viable route to optimize Cas effectors for eukaryotic systems.

Emerging evidence underscores the important role of loop engineering in Cas proteins. For example, Chen et al. developed a structure-free strategy to enhance Cas9 activity by increasing local loop flexibility (Chen et al., 2023). Using DynaMine to predict flexible regions, they introduced glycine insertions to boost conformational freedom, resulting in improved activity of GeoCas9 (Chen et al., 2023). In contrast, when applying the same predictive framework to AtCas9, the V13 and H8 loops we engineered were not predicted to be flexible and would have been deprioritized by flexibility-based criteria. This difference underscores that loop optimization can be achieved through distinct strategies. While enhancing flexibility can be effective, our approach focuses on the dynamic role of loops in mediating conformational transitions critical for allosteric regulation and catalytic activation. Rather than targeting loops with high intrinsic mobility, we prioritized regions embedded within allosteric networks, which may exhibit limited flexibility but play central roles in functional rearrangements.

Loop engineering can be implemented through point mutations, insertions, deletions, disulfide bond introduction, or segmental modification. Due to their intrinsic flexibility, loops are highly sensitive to even single-residue alterations, which can disrupt or rebuild interactions with neighboring structures. Loop engineering has traditionally focused on point-mutation, while segmental modification is rarely pursued due to the vast mutational landscape and screening burden. For example, a 10-residue loop yields 20^10^ possible variants when considering all single-residue substitutions. To circumvent this complexity, we leveraged natural sequence diversity from homologs as an evolutionary filter, enabling structurally guided substitution without exhaustive library construction. Homolog-based alignment revealed conserved rigid domains and clearly delineated loop boundaries, enabling rational loop substitution while minimizing the risk of misfolding or functional loss. A recent study demonstrated the success of this approach in enhancing IscB activity through homology-based loop engineering (Kannan et al., 2025). By narrowing the design space, this strategy increases the likelihood of preserving critical structural features.

Peripheral loops often act as temperature-sensitive switches, tuning the conformational ensemble of the enzyme under heat stress (Burns et al., 2022). In addition to conformational modulation, loops play an underappreciated role in thermal adaptation of Cas9. For example, truncating a loop in the PI domain of GeoCas9 lowered its activity threshold from 75 °C to 70 °C (Shen et al., 2024), suggesting that surface-exposed loops modulate high-temperature structural stability. Consistently, our loop engineering strategy have shown improved the activity of three thermophilic Cas9 orthologs, indicating a broader role for loop remodeling in optimizing thermal responsiveness. In the case of AtCas9, such modifications may enhance function by fine-tuning peripheral flexibility and modulating long-range conformational coupling. Extending this strategy to thermophilic orthologs, transplantation of the H8 and V13 loops into GeoCas9 and ThermoCas9 produced variants with modest activity increases at canonical PAMs but pronounced improvements at non-canonical PAM sites. These findings establish loop remodeling as a broadly applicable strategy to enhance the performance of thermophilic Cas9 orthologs, with particular advantages under suboptimal PAM conditions.

Collectively, loop regions, long considered passive connectors, emerge here as potent levers for tuning Cas9 activity. By integrating structural alignment, evolutionary filtering, and mechanistic rationale, we demonstrate that targeted loop engineering can improve editing efficiency, expand PAM compatibility, and overcome physiological constraints such as magnesium limitation. This strategy broadens the protein engineering toolkit and establishes loop design as a powerful paradigm in optimizing Cas9-based genome editing tools.

## 4 Methods

### 4.1 Plasmid construction

Plasmids for the mammalian expression of Cas9-related editors and sgRNA were cloned using the pEASY-Basic Seamless Cloning and Assembly Kit (TransGen Biotech, cat. no. CU201). A U6 promoter-driven AtCas9 sgRNA mammalian expression plasmid (designated Gcl203) was created by replacing the sgRNA scaffold in the gRNA_cloning Vector (Addgene plasmid 41824) with the AtCas9 sgRNA optimized scaffold. Expression plasmids for adenine base editors (AtCas9-ABE8e), cytosine base editors (AtCas9-BE3, rAPOBEC1-XTEN-nAtCas9-UG), and nuclease editor proteins (AtCas9 nuclease) were derived from respective AtCas9 plasmids (pAT8.5, pAT7.2, pAT301). GeoCas9 and ThermoCas9 plasmids were generated by cloning codon-optimized sequences (GenScript) into the pAT8.5, pAT7.2, and pAT301 vectors. U6 promoter-driven GeoCas9 and ThermoCas9 sgRNA plasmids (designated Gcl-Geo and Gcl-Thermo) were constructed by replacing the sgRNA scaffold sequence in the gRNA-cloning vector with GeoCas9 and ThermoCas9 sgRNA scaffolds. sgRNA sequences are listed in Supplementary Table 1.

### 4.2 Preparation of chemically modified single-guide RNA (sgRNA)

Chemically modified sgRNAs were synthesized commercially (GenScript) using an L-epeg strategy (Lei et al., 2025) to overcome length limitations inherent to conventional solid-phase synthesis. Briefly, each sgRNA was produced as two separate fragments, which were ligated *in vitro*. For each ligation, 200 pmol of sgRNA fragment 1, 200 pmol of sgRNA fragment 2, and an equimolar amount of splint DNA were combined in annealing buffer (10 mM Tris-HCl, pH 8.0, 20 mM NaCl) to a final volume of 30 μL. The mixture was heated to 70 °C for 3 min and then gradually cooled to room temperature at a rate of 0.1 °C/s. Subsequently, 4 μL of 10 × T4 RNA Ligase 2 buffer (500 mM Tris-HCl, pH 7.5, 20 mM MgCl_2_, 10 mM DTT, 4 mM ATP), 3 μL of T4 RNA Ligase 2 (1 μg/μL), and 3 μL of nuclease-free water were added. The ligation reaction was incubated at 37 °C for 1 h. To enhance yield, this annealing and ligation cycle was repeated twice more, with an additional 1.5 μL of T4 RNA Ligase 2 added in each subsequent cycle and the incubation time reduced to 30 min. Following ligation, the splint DNA was digested with DNase (Promega) at 37 °C for 30 min, and the full-length sgRNA was purified using the Monarch® RNA Cleanup Kit (New England Biolabs, NEB).

### 4.3 Preparation of AtCas9 mRNA

mRNA encoding the AtCas9-WT, Z7, or Z7-E78 base editors was prepared by *in vitro* transcription. First, linear DNA templates containing a T7 promoter and a 110-nucleotide poly(A) tail were amplified by PCR. Each 50 μL *in vitro* transcription reaction contained 0.5–1 μg of DNA template, 10 μL of 5 × T7 RNA Polymerase Reaction Buffer, 4 μL of an rNTP mix (NEB), 5 μL of 50 mM DTT, 4 μL of inorganic pyrophosphatase (Sigma), 1.25 μL of RNase Inhibitor (Promega), and 2 μL of T7 RNA polymerase. The reaction was incubated at 37 °C for 1 h, after which DNase was added to remove the DNA template. The transcribed RNA was co-transcriptionally capped using the Vaccinia Capping System (NEB) and further modified with mRNA Cap 2′-O-Methyltransferase (NEB) to generate the Cap 1 structure. Finally, the mRNA was purified using the Monarch® RNA Cleanup Kit (NEB).

### 4.4 Cell culture and transfection

HEK293T (CRL-3216) and K562 (CCL-243) cells were obtained from the American Type Culture Collection (ATCC). HEK293T cells were cultured in DMEM supplemented with 10% FBS and 1% penicillin-streptomycin. The HEK293T-EGFP cell line was generated by stable incorporation of an EF1a-EGFP vector using lentiviral transduction. K562 cells were cultured in RPMI-1640 with l-glutamine, supplemented with 10% FBS and 1% penicillin-streptomycin. Human T cells were isolated from fresh peripheral blood obtained from Saily Bio. Peripheral blood mononuclear cells (PBMCs) were separated by Ficoll density gradient centrifugation (GE Healthcare) using SepMate tubes (Stemcell Technologies). CD3^+^T cells were then purified from PBMCs with the EasySep Human T Cell Isolation Kit (Stemcell Technologies). Purified T cells were cultured in X-Vivo15 medium (Lonza) supplemented with 5% FBS (Gibco), 50 ng ml^−1^ human IL-2 (Peprotech), 10 ng ml^−1^ human IL-7 (Peprotech), and 1% penicillin–streptomycin. For activation, T cells were stimulated with CD3/CD28 Dynabeads (Thermo Fisher Scientific) at a bead-to-cell ratio of 1:3 for 3 days. After electroporation, cells were maintained in X-Vivo15 medium supplemented with 5% FBS and 100 ng ml^−1^ IL-2. Both cell types were maintained at 37 °C with 5% CO_2_ and routinely tested for mycoplasma contamination.

For transfection in HEK293T cells, 1.2 × 10^5^ cells were seeded into 48-well plates 24 h prior to transfection. A total of 450 ng of AtCas9-ABE8e, AtCas9-BE3, SpRY-ABE8e, GeoCas9-ABE8e, GeoCas9-BE3, ThermoCas9-ABE8e, or ThermoCas9-BE3 expression plasmids, along with 150 ng of sgRNA plasmids, were co-transfected into HEK293T or HEK293T-EGFP cells using Polyethylenimine (PEI)-mediated transfection. For Cas9 nuclease transfection, 400 ng of nuclease expression plasmids (pAt301) and 200 ng of spacer expression plasmids were co-transfected into HEK293T cells. K562 cells were electroporated using the Lonza 4D Nucleofector™ system, following previously established protocols. 2 × 10^6^ cells were resuspended in 20 μL of B1mix buffer (An et al., 2024; Zhang, Qiu, et al., 2023), Zhang, Zhang, et al., 2023nd 0.6 μg of AtCas9-ABE8e and 0.4 μg of sgRNA plasmids were added. Activated T cells were electroporated with 5 μg ABE mRNA and 5ug chemically modified sgRNA. The electroporation buffers were prepared as follow:

B1mix buffer: 3.6 mM KCl, 10.8 mM MgCl_2_, 62.5 mM Na_2_HPO_4_/NaH_2_PO_4_, 0.88 mM Ca(NO_3_)_2_, 10 mM sodium succinate, 18 mM mannitol, 2.8 mM inositol, 21.8 mM glucose, 2.19% GlutaMAX™, 0.55 mM sodium pyruvate, pH 7.2.

The electroporated cell mixtures were transferred into electroporation cuvettes, followed by EO138 program electroporation. Afterward, 80 μL of pre-warmed media was added to each cuvette, and the cells were allowed to recover for 10 min at 37 °C before being plated onto 48-well plates.

### 4.5 Next generation sequencing of genomic DNA samples

Two days post-transfection, cells were harvested and genomic DNA was extracted using a lysis buffer containing 10 mM Tris-HCl (pH 7.4), 0.05% SDS, and 25 μg/mL proteinase K. Cells were then incubated at 37 °C for 2 h and heat inactivated at 80 °C for 30 min. Cell lysates were used as templates for PCR amplification and subsequent sequencing. To quantify cleavage and base editing efficiencies, target genomic regions were PCR-amplified and purified using the HiPure PCR Pure Mini Kit (Magen). PCR primers are listed in Supplementary Table 1. The purified products were subjected to 2 × 150 paired-end sequencing using the MiSeq Sequencing System (Illumina). FastQC (v0.11.8 http://www.bioinformatics.babraham.ac.uk/projects/fastqc/, parameters: default) was used to evaluate raw sequencing quality. Adapter sequences and reads with Phred quality scores below 30 were trimmed. The remaining trimmed reads were aligned to the target sequences using the BWA-MEM algorithm (v0.7.17). Base editing efficiency was calculated by dividing the number of reads with converted bases by the total number of reads. Indel frequency was calculated by dividing the number of reads with indels by the total reads.

### 4.6 Protein purification

AtCas9 variants used for EMSA and thermal shift assays were purified as previously described. For EMSA experiment, nuclease dead AtCas9 (D8A H617A N640A) were purified. Briefly, codon-optimized genes encoding 6 ​× ​His-tagged AtCas9 variants were synthesized and cloned into the pET-28a (+) vector (GenScript). The recombinant plasmids were transformed into *E. coli* BL21 (DE3) cells, and protein expression was induced with 0.5 mM IPTG at 18 °C for 16 h.

Cells were harvested by centrifugation, resuspended in lysis buffer (20 mM Tris-HCl, 500 mM NaCl, pH 7.4), and lysed by sonication (Scientz). The lysate was clarified by centrifugation and filtered through a 0.22 μm membrane before purification.

Proteins were first purified using Ni-NTA Beads 6FF (Smart-Lifesciences) following batch binding procedures. Clarified lysates were incubated with pre-equilibrated Ni-NTA beads at 4 °C with gentle rotation for 1–2 h to allow binding. The beads were then washed thoroughly with wash buffer (50 mM Tris-HCl, 500 mM NaCl, 30 mM imidazole, pH 7.4) to remove non-specifically bound proteins. Target proteins were eluted using elution buffer (50 mM Tris-HCl, 500 mM NaCl, 500 mM imidazole, pH 7.4). Elution fractions were analyzed by SDS-PAGE, and those containing the target protein were pooled and concentrated using centrifugal filters (Millipore). Final protein samples were exchanged into storage buffer (50 mM Tris-HCl, 250 mM NaCl, pH 7.4) and stored at −80 °C until use.

### 4.7 RNA *In vitro* transcription

RNA was transcribed *in vitro* using DNA oligonucleotides containing a T7 promoter sequence. Transcription was performed with T7 RNA polymerase at 37 °C for 2 h. The resulting sgRNA or crRNA was purified using a column-based RNA purification kit (NEB), following the manufacturer's instructions. The DNA templates used in this study are listed in Supplementary Table S1.

### 4.8 *In vitro* cleavage assay

To evaluate the cleavage activity of AtCas9 variants, *in vitro* DNA cleavage assays were performed using linearized pCE2plasmid or PCR-amplified DNA substrates containing the target sequence. Each reaction (20 μL) contained 20 nM DNA substrate, 500 nM Cas9 protein, 1.5 μM sgRNA, and cleavage buffer (20 mM HEPES, pH 7.9, 10 mM KCl, 0.5 mM DTT, 0.1 mM EDTA, and MgCl_2_ at the indicated concentrations).

Cas9-sgRNA ribonucleoprotein (RNP) complexes were pre-assembled by incubating Cas9 protein with sgRNA at room temperature for 10 min prior to the addition of DNA substrate. Reactions were incubated at 37 °C for 5 min unless otherwise specified, and then terminated by the addition of Proteinase K (Thermo Fisher), followed by incubation at 55 °C for 10 min. DNA products were resolved on 1% agarose gels stained with ethidium bromide, and band intensities were quantified using Image Lab software. Cleavage efficiency was calculated as the ratio of cleaved DNA to total DNA.

### 4.9 Electrophoretic mobility shift assay (EMSA)

To assess protein-RNA binding, dAtCas9 was diluted to various concentrations ranging from 100 nM to 3.9 nM, and mixed with 80 nM FAM-labeled tracrRNA and crRNA in binding buffer (20 mM HEPES, pH 7.9, 10 mM KCl, 0.5 mM DTT, 0.1 mM EDTA, and MgCl_2_ at the indicated concentrations).

To assess RNP-DNA binding, dAtCas9 and sgRNA were mixed at a 1:3 M ratio in the same binding buffer. The RNP mixture was then diluted to final concentrations ranging from 100 nM to 3.9 nM and incubated at room temperature for 10 min to form RNP complexes, followed by the addition of 8 nM FAM-labeled double-stranded DNA oligonucleotides. Reactions (20 μL) were incubated at 37 °C for 1 h, then loaded onto a 4% native polyacrylamide gel pre-run in 1 × TBE at 4 °C. Electrophoresis was performed at 110 V for 40 min. Gels were imaged using a ChemiDoc MP imaging system (Bio-Rad).

### 4.10 Measurement of Protein melting temperatures

Protein thermal stability was assessed using a thermal shift assay with the GloMelt dye (Cat. No. 33021). Reactions were performed on a quantitative PCR instrument with a temperature ramp rate of 0.1 °C/s. Melting temperatures (Tm) were determined from the inflection point (peak of the derivative curve) of the fluorescence-based melting curves.

### 4.11 Structural simulations of AtCas9-RNA and AtCas9-RNA-DNA complexes

Structural simulations of WT AtCas9 and Z7 were performed using AlphaFold3, focusing on the ternary complex (AtCas9-sgRNA-DNA) with *C-myc-g41* spacer. The sgRNA sequence used for modeling was:

5′-UUAAUACCCUUCUUUCCUCCACGUCAUAGUUCCCUCACAAGCCUGAAAAGGCUUGCGAGGUUGCUAUGAUAAGGCCGAGCAACAGGCUCGUGCCGCAAAGCACUGACCCGCAUUCCAAUGAAUGCGGGUCAUCUACUUUUU-3'. DNA sequences included: Target strand: 5′-TCCCAGGGAGAGTGGAGGAAAGAAGGGTATTAA-3′ and non-target strand: 5′-TTAATACCCTTCTTTCCTCCACTCTCCCTGGGA-3'. The Z7 mutant was simulated within the ternary complex to simulate the conformational changes induced by the loop engineering.

### 4.12 Solvent-accessible surface area (SASA) analysis

SASA is a structure-based metric that indirectly reflects protein-nucleic acid interactions by measuring the extent of solvent exposure. Specifically, we computed the SASA of AtCas9s and nucleic acids individually and subtracted the SASA of the bound complex. A larger reduction in SASA indicates a greater buried surface area upon binding, which is often associated with stronger interactions (Pokala & Handel, 2004). The active state of AtCas9 was modeled using SWISS-MODEL (https://swissmodel.expasy.org/). Individual sgRNA-DNA components were extracted from AtCas9-sgRNA-DNA ternary complexes obtained from unpublished work. Ternary complexes were reassembled in PyMOL based on experimentally determined structures. SASA values for nucleic acid, protein, and the ternary complex were independently calculated using NovoPro's Protein SASA Calculator (https://www.novopro.cn/tools/calculate-solvent-accessible-surface-area.html). The interaction area was quantified by subtracting the SASA of the ternary complex from the sum of protein and nucleic acid SASA values.

### 4.13 Molecular dynamics (MD) simulations

MD simulations were performed on AtCas9(WT)-DNA-RNA complex and the AtCas9(Z7)-DNA-RNA complex using Amber20 (Lee et al., 2020. The Amber ff19SB, OL21, and OL3 forcefields were employed for protein and nucleic acid parameterization, and TIP3P water model was used to represent solvent molecules. Each protein complex was solvated in a cubic box, ensuring a minimum distance of 20 Å between the complex and the box edge. Sodium ions (Na^+^) were added to neutralize systems. A non-bonded cutoff of 1.2 nm was applied, and the Particle Mesh Ewald (PME) method was utilized for computing long-range electrostatic interactions. Pressure and temperature were maintained using Monte Carlo barostat and Langevin thermostat, with a numerical integration time step of 2 fs.

The system underwent energy minimization using the steepest descent method to resolve any spatial conflicts. Subsequently, the system was gradually heated from 100 K to 310 K. This was followed by a 100 ps constant volume (NVT) simulation, and then a 1000 ps constant pressure (NPT) simulation. During these steps, harmonic restraints with a force constant of 0.1 kcal/mol Å^−2^ were applied to the Cas9 protein and gRNA to prevent incorrect geometric configurations. The output of the NPT simulation served as the starting point for the unrestrained production simulation. This final simulation was conducted 1000 ns under constant conditions of 1 atm pressure and 310.15 K.

### 4.14 Analysis of MD simulations

To evaluate whether the engineered loops formed new atomic-level interactions with nucleic acids, we performed detailed hydrogen bond and salt bridge analyses based on the last 800 ns of 1000 ns MD trajectories, during which the systems reached equilibrium. Trajectories were recorded at 4 frames per nanosecond, yielding a total of 3200 frames for each variant. Hydrogen bonds were identified with cpptraj (donor-acceptor distance <0.35 nm; angle >135°), while salt bridges were analyzed with the MDAnalysis Python package (distance <0.4 nm between oppositely charged atoms). An interaction was defined as stable if present in more than 50% of the analyzed frames (>1600 out of 3200).

MD trajectories were analyzed using cpptraj and visualized with VMD (Humphrey et al., 1996; Roe & Cheatham, 2013). RMSD was calculated for protein heavy atoms, referencing the initial simulation structure. For RMSF calculations, trajectory data from the stable 200ns–1000ns interval were selected. Protein Cα atoms were used for RMSF calculations against the average structure, while C1' atoms were selected for nucleic acids.

Principal Component Analysis (PCA) of the MD trajectory was performed using a custom script to obtain system dynamics along Principal Component 1 (PC1), which was then visualized in VMD's Normal Mode Wizard extension (Bakan et al., 2011). Using PC1 and PC2 as reaction coordinates, the Gibbs free energy of the system was calculated based on its probability distribution along these coordinates using the Boltzmann equation, and a free energy landscape was plotted. By projecting different frames onto the PC1 and PC2 directions, the lowest energy frame and its corresponding structure were identified.

A custom script was employed to calculate the center of mass coordinates for each protein domain. This enabled the calculation and comparison of inter-domain distances in the lowest energy structures of AtCas9-WT and AtCas9-Z7.

### 4.15 GUIDE-seq

GUIDE-seq experiments were conducted by co-transfecting 5 × 10^5^ cells with 1.5 μg of pAT301 (wild-type or Z7 variant), 750 ng of Gcl203-CTNNB1-7g, and 5 pmol of double-stranded oligodeoxynucleotide (dsODN) using the Lonza 4D-Nucleofector, according to the manufacturer's instructions. Genomic DNA was harvested three days after transfection and fragmented to an average size of 200 bp using a Covaris S200 instrument. Sequencing libraries were prepared with the VAHTS Universal DNA Library Prep Kit (Vazyme) through successive steps of end repair, A-tailing, and adapter ligation. Integration sites were amplified using primers specific to the dsODN and sequenced on an Illumina platform. GUIDE-seq data analysis was performed with guideseq v1.0.2, as previously described (https://github.com/aryeelab/guideseq), using a PAM sequence of CNNA and allowing a maximum of eight mismatches during read filtering.

## CRediT authorship contribution statement

**Yue-Lin Zhang:** Writing – original draft, Validation, Investigation, Formal analysis, Conceptualization. **Dong-Chao Huang:** Visualization, Methodology, Formal analysis. **Min Duan:** Investigation. **Yu-Ming Zhang:** Investigation. **An-Hui Huang:** Investigation. **Hao Yin:** Supervision, Funding acquisition. **Ying Zhang:** Writing – review & editing, Supervision, Funding acquisition, Formal analysis, Conceptualization.

## Data availability

DNA amplicon sequencing can be accessed in the NCBI Sequence Read Archive (accession number: PRJNA1295896). Any additional information required to reanalyze the data reported in this paper is available from the lead contact upon request.

## Declaration of interests

Y.Z. holds the position of Associate Editor for Cell Insight and is blinded from peer review and decision making for the manuscript. The rest authors declared no conflict of interests.

## Appendix supplementary data

Multimedia component 1 Multimedia component 1

Fig. S1. Structural analyses of key AtCas9 mutants. a, In vitro cleavage activity of AtCas9-WT proteins at indicated temperatures under 10 ​mM magnesium concentrations. b, Analysis of protein sequence alignment and secondary structure annotation between AtCas9 and Nme1Cas9. c, Simulated structures of AtCas9 active ternary complex using Alphafold3 with engineered loops marked with red arrows. d, Comparison of WT (top) and V13 loop mutant (bottom). Engineered loop is highlighted in green and DNA is labeled yellow. The engineered loop exhibited increased stability of the secondary structure and show the interaction detail between loops and background peptide. e, Structural changes in the D1068K mutation. WT protein is colored in forest, the D1069K mutant is colored in magenta, and DNA is highlighted in yellow. f, Mutation positions and sequences of loop mutants H1-H8. g, Editing efficiencies of the indicated loop mutant for AtCas9-ABE8e at six genomic sites in HEK293T cells. n ​= ​3, data represents mean ​± ​SD from independent experimentsh, Surface charge distribution of the H8 mutant. The yellow circle marks the H8 loop, which displays increased positive charge relative to the wild type. The electrostatic surface was generated using PyMOL, with negative (red, PyMOL scale −5), positive (blue, +5), and neutral (grey) regions shown.

Multimedia component 2 Multimedia component 2

Fig. S2. In vitro DNA cleavage kinetics and nucleic acid binding activity of WT and Z7 proteins. a, In vitro DNA cleavage assay comparing WT and Z7 proteins under 10 mM Mg^2+^ conditions. Three endogenous target sites were tested: *CTNNB1-10g*, *VEGFA-11g*, and *C-myc-g41*. b, In vitro cleavage activity of WT and Z7 proteins across a range of incubation temperatures at indicated Mg^2+^ conditions. c, EMSA analysis of crRNA:tracrRNA binding by WT and Z7 proteins across a protein concentration gradient (3.9–100 nM) under 0.5 mMand 5 mM MgCl_2_ conditions. d, EMSA analysis of DNA binding by WT and Z7 RNP complexes across a concentration gradient (3.9–100 nM), under 0.5 mM and 5 mM MgCl_2_.

Fig. S3. Molecular dynamics analysis of the structural stability and flexibility of AtCas9 ternary complexesa, RMSD of protein in AtCas9-WT and AtCas9-Z7 complexes over the simulation time. **b,** Atomic-level interactions of engineered loops with nucleic acids revealed by MD simulations.

Fig. S4 | Mutational scanning of AtCas9 identifies key residues that modulate base editing activity. a, Amino acid substitution matrix summarizing the results of combinatorial mutagenesis. Beneficial mutations are highlighted in blue; substitutions at functionally important residues are shown in grey. b, Saturation mutagenesis at residue R82 in the E91 background and L544 in the E78 background. Heatmaps display editing efficiencies at the *C-myc-6g* site, along with fold changes relative to the original residues. c, A-to-G base editing efficiencies of the E5116 and E535. For c, bars represent mean ± SD from three independent biological replicates. Each dot represents one biological experiment.

Fig. S5 | Heatmaps of ABE efficiency by PAM sequencea, Key AtCas9 variants and their constituent mutations. b, Heatmaps of A-to-G conversion efficiencies for AtCas9-WT-ABE, AtCas9-Z7-ABE, and AtCas9-Z7-E78-ABE across CNNA, CNNT, CNNC, and CNNG PAM sequences.

Fig. S6 | GUIDE-seq analysis of off-target activities by AtCas9-WT and AtCas9 Z7. Genome-wide specificity profiles of AtCas9-WT and AtCas9-Z7 were assessed using the CTNNB1-7g spacer. GUIDE-seq was performed in two biological replicates with a mismatch threshold of 8. Mismatches within spacers are color-coded, and read counts for each site are shown on the right. Distinct sequencing reads corresponding to the same designated off-target site are grouped within curly brackets.

Fig. S7. Sequence alignment between thermophilic Cas9 and the mesophilic NmeCas9Corresponding H8 and V13 loop regions mapped in Nme1Cas9, AtCas9, GeoCas9, and ThermoCas9.
