# Supplementary material for: Loop engineering of AtCas9 for effective and broad genome editing
Source: Cell Insight. 2025 Oct 22;4(6):100286. doi: 10.1016/j.cellin.2025.100286 (PMC12634846; doi:10.1016/j.cellin.2025.100286)
Supplement: Multimedia component 2 [file mmc2.pdf]

Fig.S1

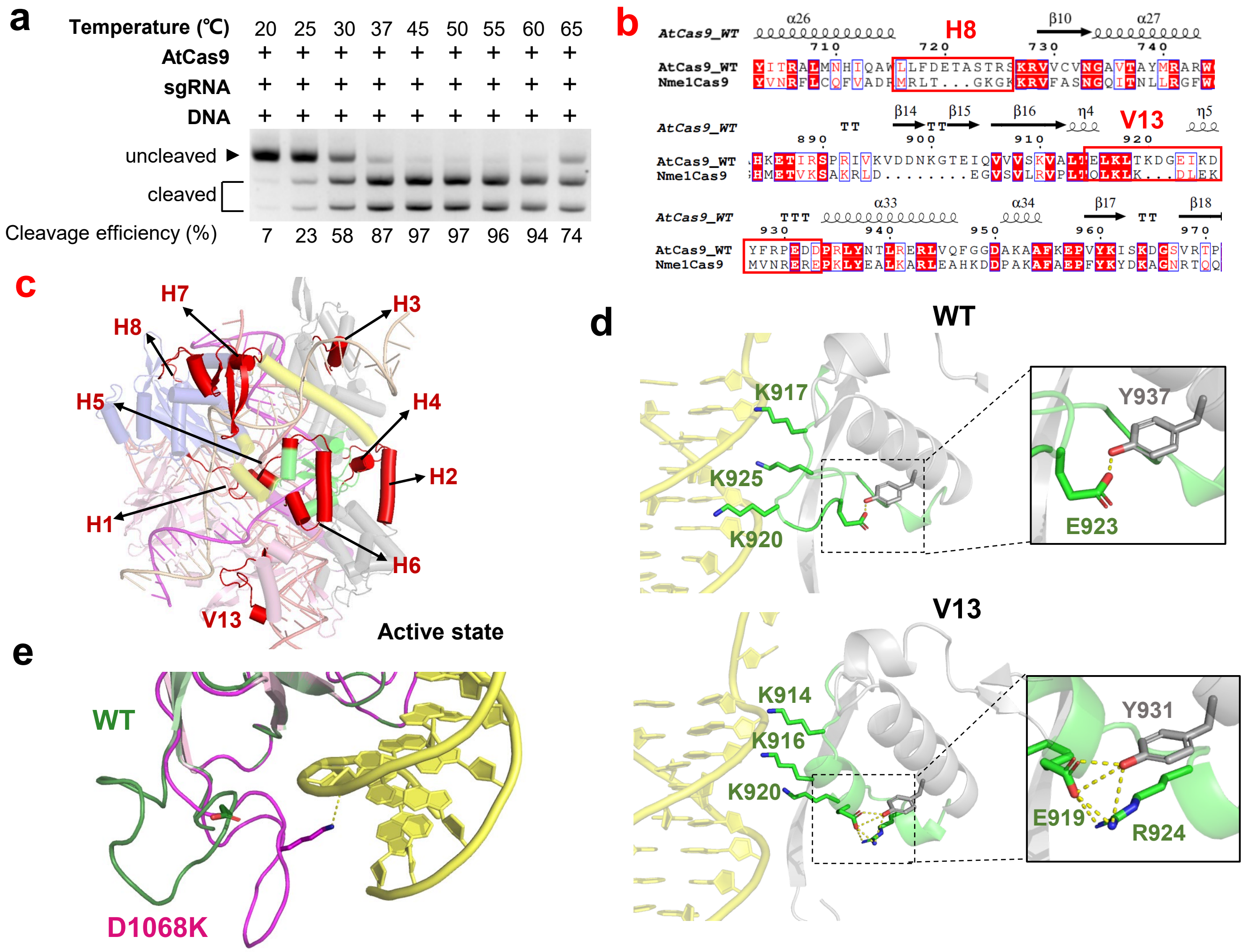

f

	At	At	Nme	Nme
H1	40-56	IFDKAEDSKTGASLALP	45-61	VFERAEVPKTGDSLAMA
H2	141-166	SEIKDSDAGKLLKAVQENENLMQS	147-172	NEGETADKELGALLKGVAGNAHALQT
H3	369-383	LNRSRNKTEDTVFV	339-348	KDNAEASTLM
H4	602-611	LDVTRLFEP	573-581	INLGRLNEK
H5	622-630	GISFDDSYD	593-602	QRTRDDSFN
H6	646-674	RTPLEYLRDKPEQKAKFIALVESIPLSQK	617-647	QTPYEYFNGKDNSREWQEFKARVETSRFPRS
H7	782-832	RNALADRNRYVQQVSKSEGITQYVDKETGEVFTWESFDERKFLPNEPLE	748-775	EMNAFDGKTIDKETGEVLHQKTHFPQ
H8	717-727	LFDETASTRS	686-692	RLTGKGK

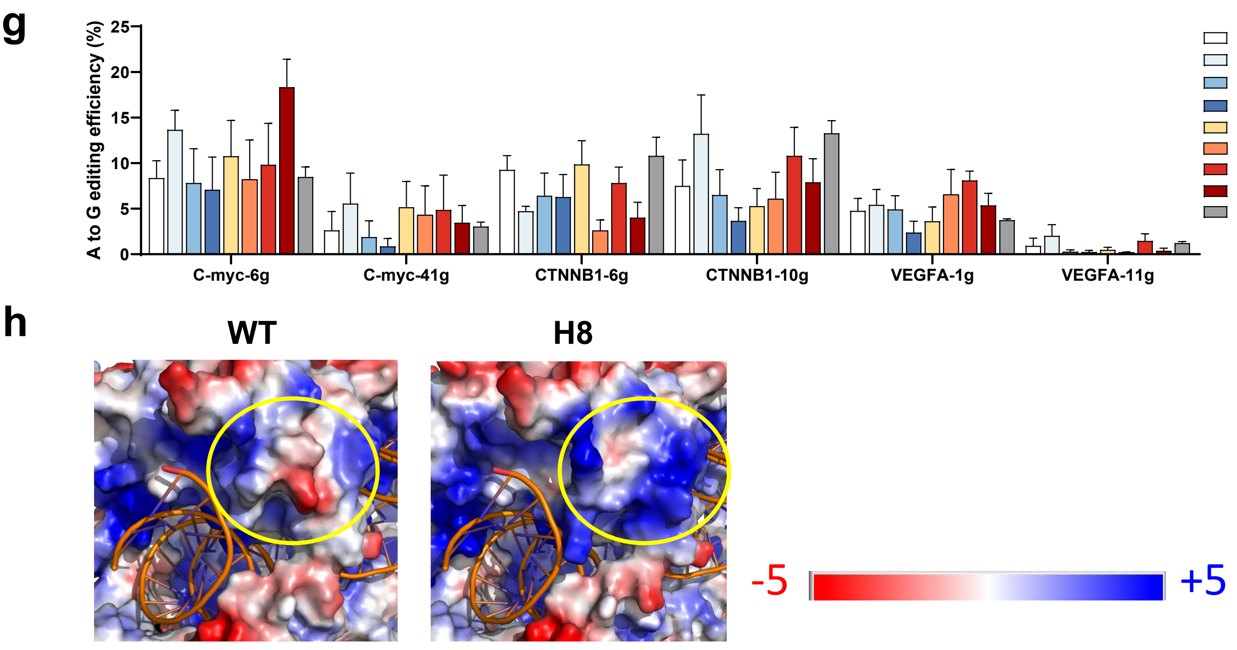

Fig.S2

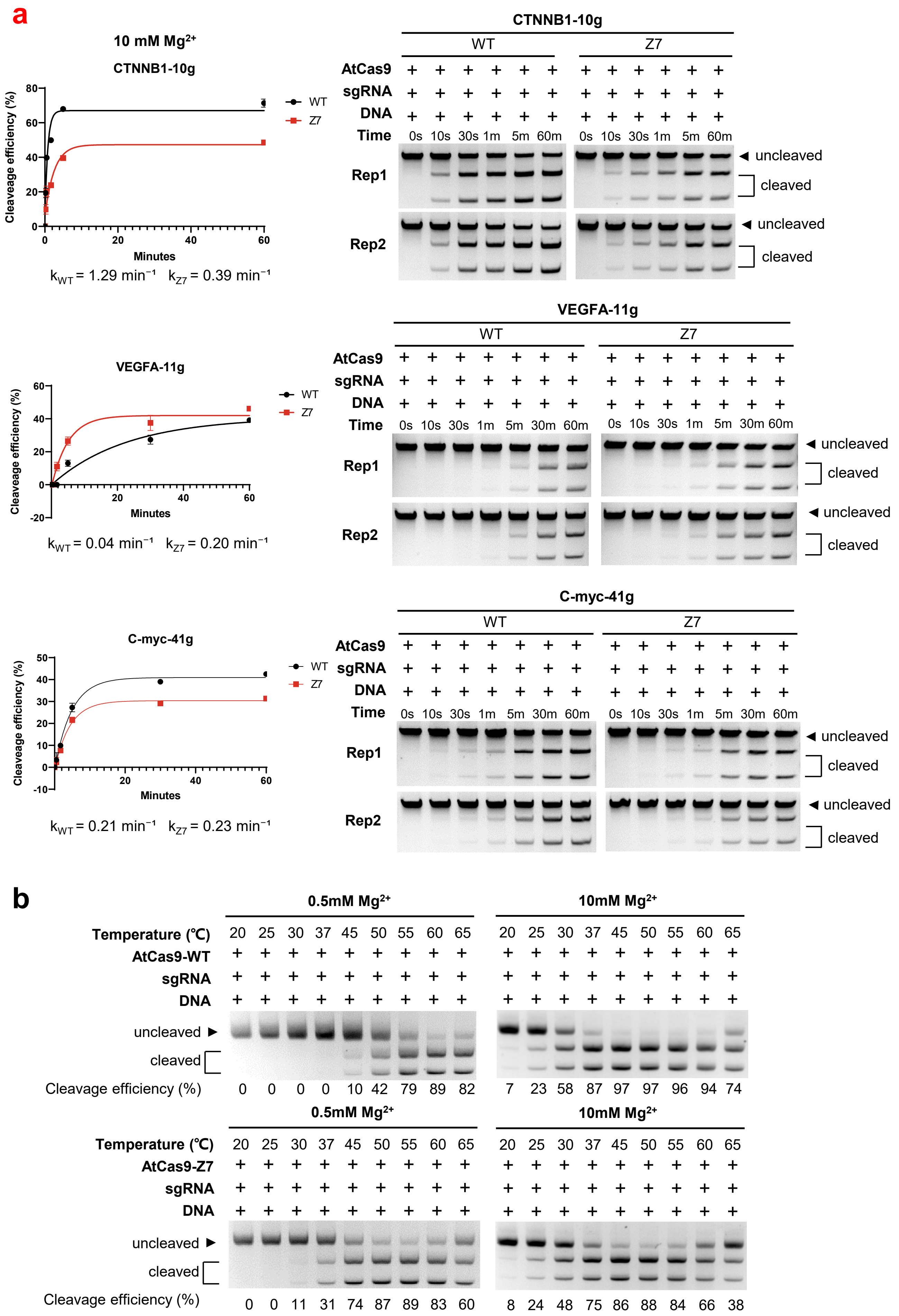
a
10 mM Mg2+
CTNNB1-10g
Cleavage efficiency (%)
Minutes
WT
Z7
kWT = 1.29 min−1 kZ7 = 0.39 min−1
CTNNB1-10g
WT
Z7
AtCas9
sgRNA
DNA
Time
0s 10s 30s 1m 5m 60m
Rep1
Rep2
uncleaved
cleaved
VEGFA-11g
kWT = 0.04 min−1 kZ7 = 0.20 min−1
0s 10s 30s 1m 5m 30m 60m
C-myc-41g
kWT = 0.21 min−1 kZ7 = 0.23 min−1
b
0.5mM Mg2+
10mM Mg2+
Temperature (℃)
20 25 30 37 45 50 55 60 65
AtCas9-WT
uncleaved
cleaved
Cleavage efficiency (%)
0 0 0 0 10 42 79 89 82
7 23 58 87 97 97 96 94 74
AtCas9-Z7
0 0 11 31 74 87 89 83 60
8 24 48 75 86 88 84 66 38

Fig.S2

c

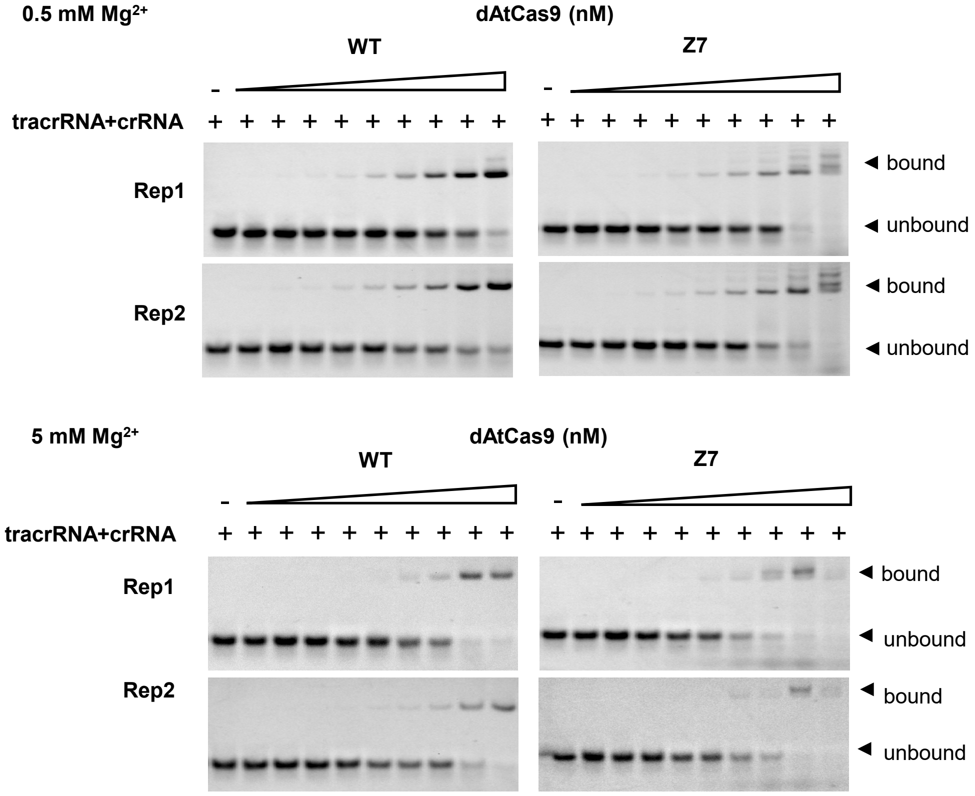
0.5 mM Mg2+
dAtCas9 (nM)
WT
Z7
tracrRNA+crRNA
Rep1
Rep2
bound
unbound
5 mM Mg2+
dAtCas9 (nM)
WT
Z7
tracrRNA+crRNA
Rep1
Rep2
bound
unbound

d

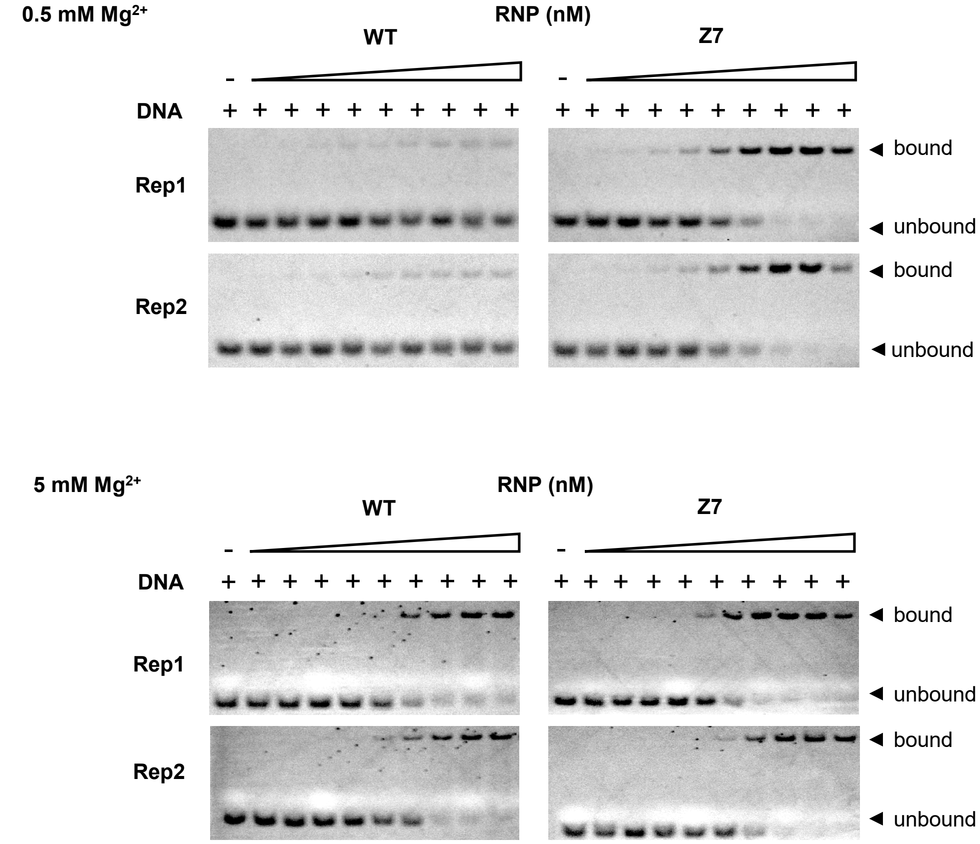
0.5 mM Mg2+
RNP (nM)
WT
Z7
DNA
Rep1
Rep2
bound
unbound
5 mM Mg2+
RNP (nM)
WT
Z7
DNA
Rep1
Rep2
bound
unbound

Fig.S3

a

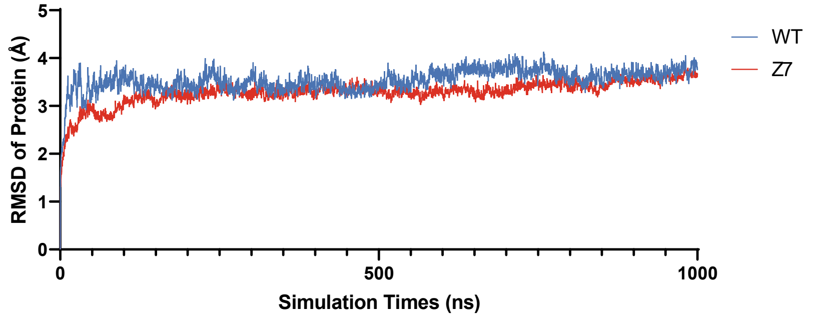

b

	WT	H8
Hydrogen bonds	None	K724–DA (DNA)
Salt bridges	None	None

	WT	V13
Hydrogen bonds	None	N923–U
Salt bridges	K925-DC; K917-DA	K914–DG; K916–DT ; K920–DC

Fig.S4

a

Varients	Spacers				Amino Acids																		
	EGFP-17g	EGFP-3g	EMX1-1g	CTNNB1-7g	48	56	82	83	198	259	477	497	504	544	557	607	608	610	615	622	623	1068	1087
WT	13.6	3.8	48.0	20.4	K	P	E	Q	N	D	E	P	T	L	N	L	F	P	V	G	G	D	G
VZH22	27.6																N	K				K	
E29	34.2											R					N	K				K	
E52	46.1	11.0	60.4				R					R					N	K				K	
E53	35.0											R	R				N	K				K	
E54	36.8											R					N	K				K	S
E55	37.9						R										N	K				K	S
E56	37.2						R						R				N	K				K	S
E60		22.7	63.7		W		R					R					N	K				K	
E61		23.7	61.3		Y		R					R					N	K				K	
E65		20.2				A	R					R					N	K				K	
E79			62.4		W	A	R					R					N	K				K	
E80			60.5		W		R	R				R					N	K				K	
E81			50.2		W		R			R		R					N	K				K	
E82			54.4		W		R				R	R					N	K				K	
E83			68.2	42.0	Y	A	R					R					N	K				K	
E84			61.8		Y		R	R				R					N	K				K	
E85			61.8		Y		R			R		R					N	K				K	
E86			63.7		Y		R				R	R					N	K				K	
E77				45.7	Y	A	R					R		R			N	K				K	
E78				48.1	Y	A	R					R			R		N	K				K	
E90				33.2	Y	A	R					R				R	N	K				K	
E91				43.9	Y	A	R					R					N	K	R			K	
E92				43.3	Y	A	R					R					N	K		R		K	
E93				29.9	Y	A	R					R					N	K		S	R	K	
E103				28.5	Y	A	R		R			R					N	K	R			K	
E105				24.6	Y	A	R				R	R					N	K	R			K	
E106				25.8	Y	A	R					R		R			N	K	R			K	
E107				26.9	Y	A	R					R			R		N	K	R			K	

b

Nonpolar aliphatic — Polar uncharged — Bulky aromatic — Polar charged

	G	A	V	L	M	I	S	T	C	P	N	Q	F	Y	W	K	R	H	D	E
E91_R82	0.99	0.94	0.86	0	0.98	1.06	0.90	1.05	0.86	0.79	0.90	0.96	0.96	1.01	0.92	0.88	1.00	0.85	0.92	0.82
E78_L544	1.17	0.90	1.10	1.00	0.01	0.72	1.03	1.02	1.09	1.15	1.10	1.00	0.34	0.88	0.95	0.93	1.02	0.95	1.18	1.19

E5116

E535

Scale: 1.0, 0.5, 0

c

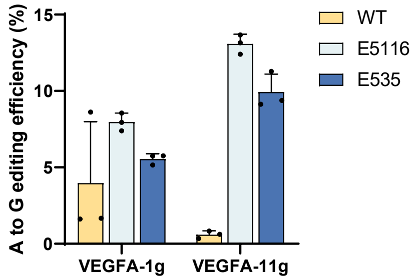

Fig.S5

a

	48	56	82	497	557	608	610	615	717-727	915-933	1068
	K	P	E	P	N	F	P	V	LFDETASTRS	ELKLTKDGEIKDYFRPEDD	D
V13										QLKLKDLEKMVNRERE	
Z7									RLTGKGK	QLKLKDLEKMVNRERE	K
E78	Y	A	R	R	R	N	K				K
E5116	Y	A		R		N	K	R			K
Z7-E78	Y	A	R	R	R	N	K		RLTGKGK	QLKLKDLEKMVNRERE	K

b

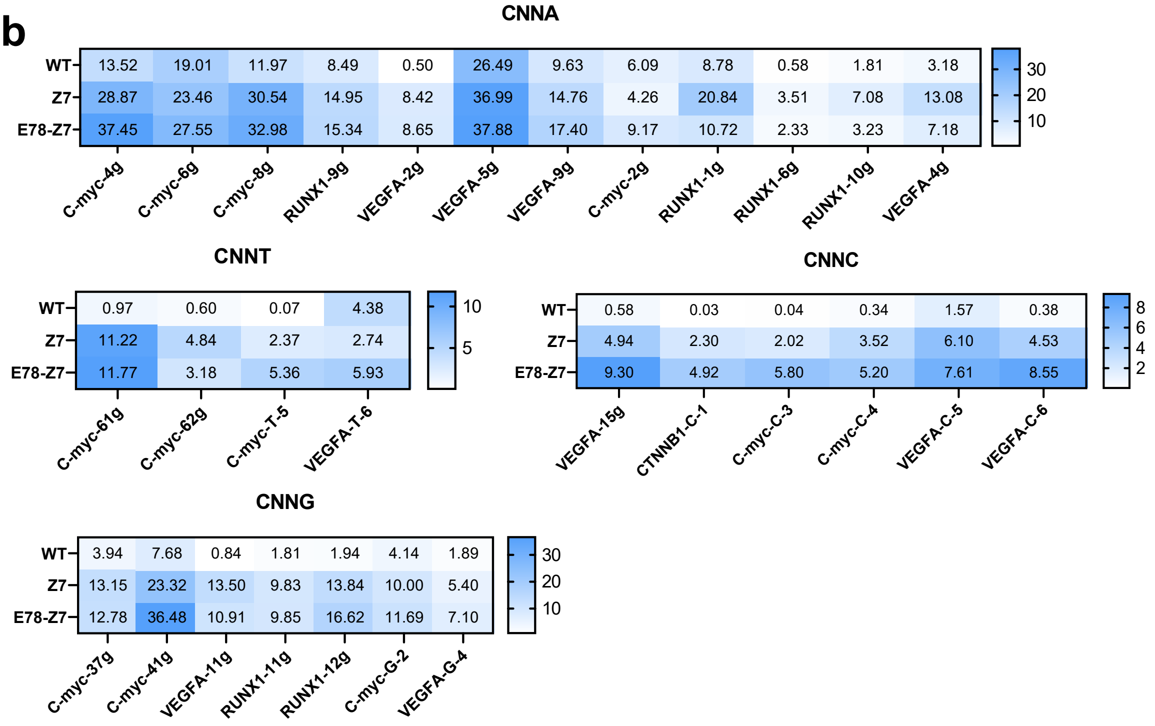

Fig.S6

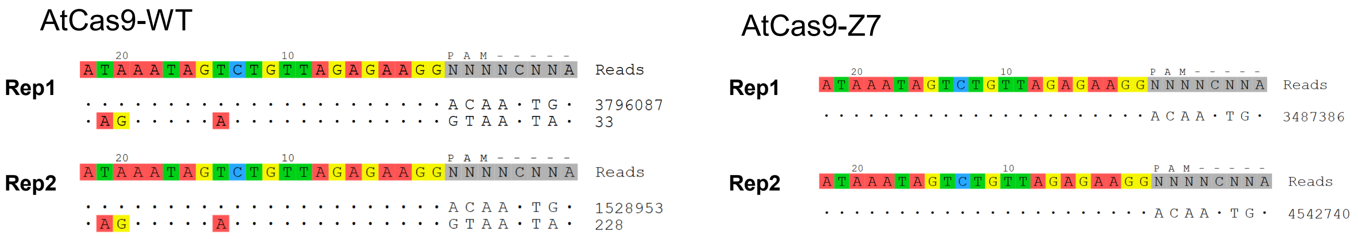

AtCas9-WT
Rep1
20
10
PAM
ATAAATAGTCTGTTAGAGAAGGNNNNCNNA
Reads
ACAA·TG·
3796087
·AG······A·
GTAA·TA·
33
Rep2
1528953
228
AtCas9-Z7
3487386
4542740

Fig.S7

```
                                             H8
Nme1Cas9       DEDGFKERNLNDTRYVNRFLCQFVADRMRLTGK---GKKRVFASNGQITNLLRGFWGLRK    714
AtCas9_WT      EQEGFRERNLSDTRYITRALMNHIQAWLLFDETASTRSKRVVCVNGAVTAYMRARWGLTK    748
GeoCas9        EETEFKNRNLNDTRYISRFFANFIREHLKFAESD--DKQKVYTVNGRVTAHLRSRWEFNK    711
ThermoCas9     EENEFKNRNLNDTRYISRFLANFIREHLKFADSD--DKQKVYTVNGRITAHLRSRWNFNK    711
               ::  *::***.****:.* : :.:   : :  .    .::*   ** :*  :*. * : *

                     V13
Nme1Cas9       LTQLKLK---DLEKMVNREREPKLYEALKARLEAHKDDPAKAFAEPFYKYDKAGNRTQQV    920
AtCas9_WT      LTELKLTKDGEIKDYFRPEDDPRLYNTLRERLVQFGGDAKAAFKEPVYKISKDGSVRTPV    972
GeoCas9        LSEIKLDASGHFP-MYGKESDPRTYEAIRQRLLEHNNDPKKAFQEPLYKPKKNGEPGPVI    899
ThermoCas9     LSEIQLDKTGHFP-MYGKESDPRTYEAIRQRLLEHNNDPKKAFQEPLYKPKKNGELGPII    899
               *::::*    .:      * :*: *:::: **  . .*   ** **.** .* *.    :
```
